# Multiple Imputation for General Missing Data Patterns in the Presence of High-dimensional Data

**DOI:** 10.1038/srep21689

**Published:** 2016-02-12

**Authors:** Yi Deng, Changgee Chang, Moges Seyoum Ido, Qi Long

**Affiliations:** 1 Department of Biostatistics and Bioinformatics, Emory University, Atlanta, 30322, USA; 2 Georgia Department of Public Health, 30303, USA

## Abstract

Multiple imputation (MI) has been widely used for handling missing data in biomedical research. In the presence of high-dimensional data, regularized regression has been used as a natural strategy for building imputation models, but limited research has been conducted for handling general missing data patterns where multiple variables have missing values. Using the idea of multiple imputation by chained equations (MICE), we investigate two approaches of using regularized regression to impute missing values of high-dimensional data that can handle general missing data patterns. We compare our MICE methods with several existing imputation methods in simulation studies. Our simulation results demonstrate the superiority of the proposed MICE approach based on an indirect use of regularized regression in terms of bias. We further illustrate the proposed methods using two data examples.

Missing data are often encountered for various reasons in biomedical research and present challenges for data analysis. It is well known that inadequate handling of missing data may lead to biased estimation and inference. A number of statistical methods have been developed for handling missing data. Largely due to its ease of use, multiple imputation (MI)^1^,^2^ has been arguably the most popular method for handling missing data in practice. The basic idea underlying MI is to replace each missing data point with a set of values generated from its predictive distribution given observed data and to generate multiply imputed datasets to account for uncertainty of imputation. Each imputed data set is then analyzed separately using standard complete-data analysis methods and the results are combined across all imputed data sets using Rubin’s rule^1^,^2^. MI can be readily conducted using available software packages^3^,^4^,^5^ in a wide range of situations and has been investigated extensively in many settings^6^,^7^,^8^,^9^,^10^,^11^,^12^. Most of the existing MI methods rely on the assumption of *missingness at random* (MAR)^2^, i.e., missingness only depends on observed data; our current work also focuses on MAR. In recent years, the amount of data has increased considerably in many applications such as omic data and electronic health record data. In particular, the high dimensions in omic data may cause serious problems to MI in terms of applicability and accuracy. In what follows, we first describe some challenges of MI in the presence of high-dimensional data and explain why regularized regressions are suitable in this setting, and then review existing MI methods for general missing data patterns and propose their extensions for high-dimensional data.

Advances in technologies have led to collection of high-dimensional data such as omics data in many biomedical studies where the number of variables is very large and missing data are often present. Such high-dimensional data present unique challenges to MI. When conducting MI, Meng^13^ suggested imputation models be as general as data allow them to be, in order to accommodate a wide range of statistical analyses that may be conducted using multiply imputed data sets. However, in the presence of high-dimensional data, it is often infeasible to include all variables in an imputation model. As such, machine learning and model trimming techniques have been used in building imputation models in these settings. Stekhoven *et al*.^14^ proposed a random forest-based algorithm for missing data imputation called missForest. Random forest utilizes bootstrap aggregation of multiple regression trees to reduce the risk of overfitting, and combines the predictions from trees to improve accuracy of predictions^15^. Shah *et al*.^16^ suggested a variant of missForest and compared it to parametric imputation methods. They showed that their proposed random forest imputation method was more efficient and produced narrower confidence intervals than standard MI methods. Liao *et al*.^17^ developed four variations of K-nearest-neighbor (KNN) imputation methods. However, these methods are improper in the sense of Rubin (1987)^1^ since they do not adequately account for the uncertainty of estimating parameters in the imputation models. Improper imputation may lead to biased parameter estimates and inference in subsequent analyses. In addition, KNN methods are known to suffer from the curse of dimensionality^18^,^19^ and hence may not be suitable for high-dimensional data. Apart from random forest and KNN, regularized regression, which allows for simultaneous parameter estimation and variable selection, presents another option for building imputation models in the presence of high-dimensional data. The basic idea of regularized regression is to minimize the loss function of a regression, subject to some penalties. Different penalty specifications give rise to various regularized regression methods. Zhao and Long (2013)^20^ investigated the use of regularized regression for MI including lasso^21^, elastic net^22^ (EN), and adaptive lasso^23^ (Alasso). They also developed MI using a Bayesian lasso approach. However, they focused on the setting where only one variable has missing values. There has been limited work on MI methods for general missing data patterns where multiple variables have missing values in the presence of high-dimensional data.

To handle general missing data patterns, there are two MI approaches, one based on joint modeling (JM)^24^ and the other based on fully conditional specifications, the latter of which is also known as multiple imputation by chained equations (MICE) and has been implemented independently by van Buuren *et al*. (2011)^3^ and Raghunathan *et al*. (1996)^25^. While JM has strong theoretical justifications and works reasonably well for low-dimensional data, its performance deteriorates as the data dimension increases^26^ and it is difficult to extend to high-dimensional data. MICE involves specifying a set of univariate imputation models. Since each imputation model is specified for one partially observed variable conditional on the other variables, it simplifies the modeling process. While MICE lacks theoretical justifications except for some special cases^27^,^28^, it has been shown to achieve satisfactory performance in extensive numerical studies and empirical examples. White *et al*. (2011)^29^ provides a nice review and guidance for MICE. It is worth mentioning that standard MICE methods cannot handle high-dimensional data. For example, the MICE algorithms proposed by van Buuren *et al*.^3^ and Su *et al*.^5^ cannot handle the prostate cancer data used in our data analysis and the high-dimensional data generated in our simulations. As such, we focus on extending MICE to high-dimensional data settings for handling general missing data patterns.

## Methodology

Suppose that our data set **Z** has *p* variables, **z**_1_, …, **z**_*p*_. Without loss of generality, we assume that the first *l* (*l* ≤ *p*) variables contain missing values. Suppose the data consist of *n* observations and we have *r*_*j*_ observed values in variable **z**_*j*_. We denote the observed components and missing components for variable *j* by **z**_*j*,*obs*_ and **z**_*j*,*mis*_. Let 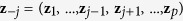 be the collection of the *p* − 1 variables in **Z** except **z**_*j*_. Let **z**_−*j*,*obs*_ and **z**_−*j*,*mis*_ denote the two components of **z**_−*j*_ corresponding to the complement data of **z**_*j*,*obs*_ and **z**_*j*,*mis*_.

### Multiple imputation by chained equations

Let the hypothetically complete data **Z** be a partially observed random draw from a multivariate distribution 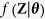. We assume that the multivariate distribution of **Z** is completely specified by the unknown parameters ***θ***. The standard MICE algorithm obtains a posterior distribution of ***θ*** by sampling iteratively from conditional distributions of the form 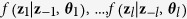. Note that the parameters ***θ***_1_, …, ***θ***_*l*_ are specific to the conditional densities, which might not determine the unique ‘true’ joint distribution 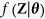.

To be specific, MICE starts with a simple imputation, such as imputing the mean, for every missing value in the data set. Initial values are denoted by 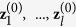. Then given values 
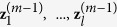
 at iteration 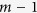, for variable 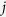, new parameter estimates 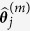 of the next iteration are generated from 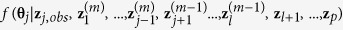 through a regression model. Then the missing values 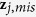 for 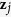 are replaced with predicted values from the regression model with model parameter 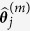. Note that when 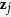 is subsequently used as a predictor in the regression model for other variables that have missing values, both the observed and predicted values are used. These steps are repeated for each variable with missing values, that is, **z**_1_ to **z**_*l*_. Each iteration entails cycling through imputing **z**_1_ to **z**_*l*_. At the end of each iteration, all missing values are replaced by the predictions from regression models that expose the relationships observed in the data. We then repeat the procedures iteratively until convergence. The complete algorithm can be described as follows:


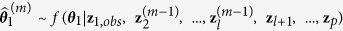



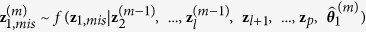



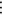



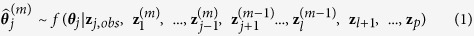



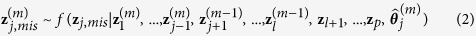



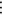


Note that while the observed data **z**_*obs*_ do not change in the iterative updating procedure, the missing data **z**_*mis*_ do change from one iteration to another. After convergence, the last 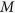 imputed data sets after appropriate thinning are chosen for subsequent standard complete-data analysis.

In the case of high-dimensional data, where *p* > *r*_*j*_ or *p* ≈ *r*_*j*_, it is not feasible to fit the imputation model (1) using traditional regressions. In the following two subsections, we provide details of two approaches to apply regularized regression techniques in the presence of high-dimensional data for general missing data patterns.

### Direct use of regularized regression for multiple imputation

For variable **z**_*j*_, our goal is to fit the imputation model (1) using *r*_*j*_ cases with observed **z**_*j*_. Assuming that *q*_*j*_ variables in **z**_−*j*,*obs*_ are associated with **z**_*j*,*obs*_, we denote this set of 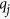 variables by 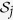, which is also known as the true active set. We define the subset of predictors that are selected to impute 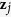 as the active set by 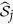, and denote the corresponding design matrix as 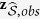.We first consider an approach where a regularization method is used to conduct both model trimming and parameter estimation and a bootstrap step is incorporated to simulate random draws from 
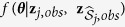
. This approach is referred to as MICE through the direct use of regularized regression (MICE-DURR). The purpose of the boostrap is to accommodate sampling variation in estimating population regression parameters, which is part of ensuring that imputations are proper^16^. In the 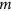-th iteration and for variable 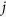, (*j* = 1, …, *l*), define 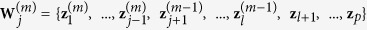. Denote by 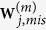 the component of 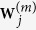 corresponding to 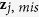. The algorithm can be described as follows:

1. Generate a bootstrap data set 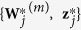 of size 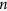 by randomly drawing 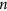 observations from 
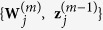
 with replacement. Denote the observed values of 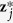 by 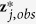 and the corresponding component of 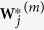 by 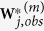.
2. Regarding 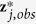 as the outcome and 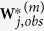 as predictors, use a regularized regression method to fit the model and obtain 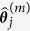. Note that 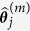 is considered a random draw from 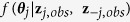.
3. Predict 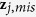 with 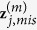 by drawing randomly from the predictive distribution 
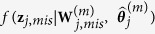
, noting that imputation is conducted on the original data set 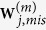, not the bootstrap data set 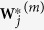.

We conduct the above procedure for 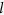 variables that have missing values in one iteration and repeat iteratively to obtain 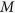 imputed data sets. Subsequently, standard complete-data analysis can be applied to each one of the 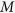 imputed data sets.

We make our approach clear by linking the above three steps to the MICE algorithm. In the first step, we bootstrap the data from the last iteration to ensure that the following imputations are proper. In the second step, we use regularized regressions to fit model (1) and obtain an estimate of ***θ***_*j*_. Then, we use this estimate to predict the missing values from the model (2). Details of MICE-DURR for three types of data can be found as Supplementary Method S1 online.

### Indirect use of regularized regression for multiple imputation

MICE-DURR uses regularized regression for both model trimming and parameter estimation. An alternative approach to MICE-DURR is to use a regularization method for model trimming only and then followed by a standard multiple imputation procedure using the estimated active set (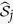), say, through a maximum likelihood inference procedure. We refer to this approach as MICE through the indirect use of regularized regression (MICE-IURR). Suppose 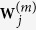 is defined as above. Denote by 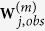 the component of 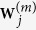 corresponding to 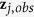. At the 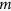-th iteration and for variable 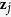, the algorithm of the MICE-IURR approach is as follows:

1. We use a regularized regression method to fit a multiple linear regression model regarding 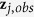 as the outcome variable and 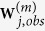 as the predictor variable, and identify the active set, 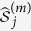. Let 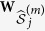 denote the subset of 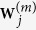 that only contains the active set. Correspondingly, denote two components of 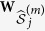 by 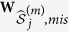 and 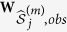.
2. Approximate the distribution of 
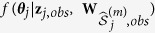
 by using a standard inference procedure such as maximum likelihood.
3. Predict 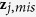: randomly draw 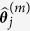 from 
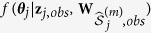
 and subsequently predict 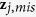 with 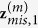 by drawing randomly from the predictive distribution 
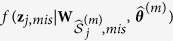
.

These three steps are conducted iteratively until convergence. We obtain the last 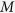 imputed data sets for the following analyses. In the third step, instead of fixing one 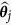 for all iterations, we randomly draw 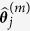 from the distribution and use it to predict 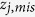 at each iteration. This strategy can guarantee that our imputations are proper^30^. Details of MICE-IURR for three types of data can be found as Supplementary Method S2 online.

## Simulation Studies

Extensive simulations are conducted to evaluate the performance of the two proposed methods MICE-DURR and MICE-IURR in comparison with the standard MICE and several other existing methods under general missing data patterns. For MICE-DURR and MICE-IURR, we consider three regularization methods, namely, lasso, EN and Alasso. We summarize the simulation results over 200 Monte Carlo (MC) data sets.

The setup of the simulations is similar to what was used in Zhao and Long (2013). Specifically, each MC data set has a sample size of 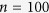 and includes 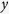, the fully observed outcome variable, and 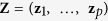, the set of predictors and auxiliary variables. We consider settings with 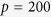 and 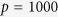. We consider 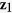, 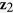, and 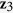 having missing values, which follow a general missing data pattern. We first generate 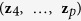 from a multivariate normal distribution with mean 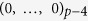 and a first order autoregressive covariance matrix with autocorrelation 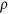 varying as 0, 0.1, 0.5, and 0.9. Given 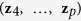, variables 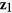, 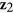, and 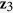 are generated independently from a normal distribution 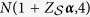, where 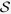 represents the common true active set with a cardinality of 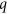 for all variables with missing values. We further consider settings where 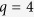 and 20, and 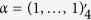 for 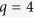; 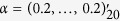 for 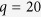. For 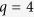 and 20, the corresponding true active set 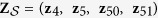 and 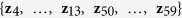. Given **Z**, the outcome variable **y** is generated from 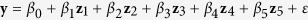, where 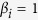, and 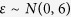 is random noise and independent of 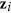. Missing values are created in 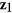, 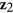, and 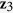 using the following logit models for the corresponding missing indicators, 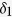, 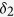, and 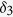, 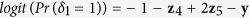, 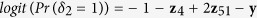, and 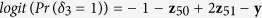, resulting in approximately 40% of observations having missing values.

We compare our proposed MICE-DURR and MICE-IURR with the random forest imputation method (MICE-RF)^16^ and two KNN imputation methods^17^, one by the nearest variables (KNN-V) and the other by the nearest subjects (KNN-S). When applying MICE-RF, KNN-V, and KNN-S, the corresponding R packages returned errors when the incomplete dataset contains large number of variables (i.e. 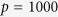). As a result, these three methods are only applied to the setting of 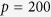. Since the standard MI method as implemented in the R package mice is not directly applicable to the setting of 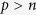, we consider a standard MI approach that uses the true active set 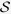 plus **y** to impute 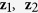 and 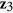, denoted by MI-true. Of note, MI-true is not applicable in practice since the true active set is in general unknown.

Following Shah *et al*.^16^, 10 imputed datasets are generated using each MI method; then a linear regression model is fitted to regress **y** on (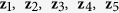) in each imputed data set and Rubin’s rule is applied to obtain 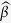 and their SEs. Consistent with the recommendations in the literature^3^,^29^, we find in our numerical studies that imputed values using all the MI methods are fairly stable after 10 iterations and hence fix the number of iterations to 20. To benchmark bias and loss of efficiency in parameter estimation, two additional approaches that do not involve imputations are also included: a gold standard (GS) method that uses the underlying complete data before missing data are generated, and a complete-case analysis (CC) method that uses only complete-cases for which all the variables are observed^2^. We calculate the following measures to summarize the simulation results for 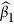, 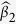, and 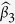: mean bias, mean standard error (SE), Monte Carlo standard deviation (SD), mean square error (MSE) and coverage rate of the 95% confidence interval (CR).

Tables 1, 2, 3 summarize the results for 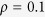, 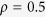 and 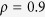, respectively. Within each table different methods are compared and the effects of the cardinality of the true active set 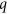 and dimension 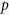 are evaluated with the correlation 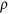 fixed. In all scenarios, GS and MI-true, neither of which is applicable in real data, lead to negligible bias and their CRs are close to the nominal level, whereas the complete-case analysis and the existing MI methods including MICE-RF, KNN-V and KNN-S lead to substantial bias. In particular, MICE-RF, with a large bias, tends to obtain a large coverage rate close to 1. KNN-V and KNN-S, on the other hand, impute the missing values only once and exhibit under-coverage of 95% CI, likely a result of improper imputation. MICE-DURR performs poorly with substantial bias in our settings with general missing data patterns. Of note, MICE-DURR was shown in Zhao and Long (2013)^20^ to improve the accuracy of the estimate in the simulation settings where only one variable has missing values. In comparison, the MICE-IURR approach achieves better performance–in terms of bias–than the other imputation methods except for MI-true. In all settings, the MICE-IURR method using lasso or EN exhibits small to negligible bias, similar to MI-true. When 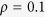, the biases and MSEs for MICE-RF, KNN-V, and MICE-DURR decrease as 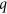 increases, whereas the performance of KNN-S deteriorates. The MICE-IURR methods tend to give fairly stable results as 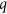 changes. When 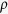 and 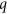 are fixed, the results of MICE-DURR and MICE-IURR with 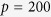 are very similar compared with the results with 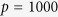.

Compared with Tables 1, 2, 3 show similar patterns in terms of comparisons between the imputation methods. Among the three MICE-IURR algorithms, Alasso tends to underperform lasso and EN when 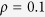 (Table 1), but not so when 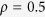 and 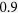 (Tables 2 and 3). In addition, when 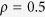, the biases and MSEs decrease for MICE-IURR using lasso and EN and increase for MICE-IURR using Alasso, as 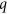 increases from 200–1000.

## Data Examples

We illustrate the proposed methods using two data examples.

### Georgia stroke registry data

Stroke is the fifth leading cause of death in the United States and a major cause of severe long-term disability. The Georgia Coverdell Acute Stroke Registry (GCASR) program is funded by Centers for Disease Control Paul S. Coverdell National Acute Stroke Registry cooperative agreement to improve the care of acute stroke patients in the pre-hospital and hospital settings. In late 2005, 26 hospitals initially participated in GCASR program and this number increased to 66 in 2013, which covered nearly 80% of acute stroke admissions in Georgia. Intravenous (IV) tissue-plasminogen activator (tPA) improves the outcomes of acute ischemic stroke patients, and brain imaging is a critical step in determining the use of IV tPA. Time plays a significant role in determining patients’ eligibility for IV tPA and their prognosis. The American Heart and American Stroke Association and CDC set a goal that hospitals should complete imaging within 25 minutes of patients arrival to a hospital. The objective of this study, thus, is to identify the factors that might be associated with hospital arrival-to-imaging time. GCASR collected data on 86,322 clinically diagnosed acute stroke admissions between 2005 and 2013. The registry has 203 data elements of which 121 (60%) have missing values, attributed to lack of answers, service not provided, poor documentation and data abstraction or ineligibility of a patient to a specific care. The extent of missingness varies from 0.01–28.72%.

In this analysis, we consider arrival-to-CT time the outcome and the other 13 variables the predictors. These 13 variables of interest can be classified into two categories: patient-related variables such as age, gender, health insurance, and medical history; pre-hospital-related variables such as EMS notification. Only gender, age and race are fully observed among 13 variables. A CC analysis is conducted which uses only 15% of the original subjects after the removal of incomplete cases. In addition, MI methods are also used. We first remove variables that have missing rate greater than 40% and the remaining variables are used to impute the missing values of partially observed variables that are of interest. After imputations, each imputed datasets of 86,322 subjects are used to fit the regression models separately and results are combined by Robin’s rules. We use a straightforward and popular strategy to handle skip pattern: first treat skipped item as missing data and impute them along with other real missing values, then restore the imputed values for skipped items back to skips in the imputed data sets to preserve skip patterns. We apply five MI methods, namely, the MICE method proposed by van Buuren *et al*.^3^ (mice), the MI method proposed by Su *et al*.^5^ (mi), the random forest MICE method proposed by Shah *et al*.^16^ (MICE-RF), and our MICE-DURR and MICE-IURR methods. When applying KNN-V and KNN-S, the R software returned errors. Thus, KNN-V and KNN-S are not included in this data example.

Table 4 provides the results from our data analyses. In the CC analysis, only NIH stroke score and race are shown to be associated with the arrival-to-CT time. The results from all five MI methods are similar in terms of the p-value and the direction of the association. By comparison, while only 2 variables are shown to be statistically significant in the CC analysis, this number increases to 11, 11, 10, 9 and 9 for mi, mice, MICE-RF, MICE-DURR, and MICE-IURR, respectively. For example, after adjusting for other variables, the mean arrival-to-CT time in patients that arrive during the day time (Day) was 18.4 minutes shorter than that in patients arriving at night (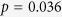) based on MICE-IURR imputation. Health insurance and three variables about history of diseases become statistically significant after we apply the MI methods. However, NIH stroke score and race, which are shown to be statistically significant by CC analysis, turn out to be not significant by MICE-DURR and MICE-IURR.

### Prostate cancer data

The second data set is from a prostate cancer study (GEO GDS3289). It contains 99 samples, including 34 benign epithelium samples and 65 non-benign samples, with 20,000 genomic biomarkers. Missing values are present for 17,893 biomarkers, nearly 89% of all genomic biomarkers in this data set. In this analysis, we consider a binary outcome 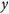, defined as 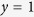 if it is a benign sample and 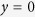 if otherwise, and test whether some genomic biomarkers are associated with the outcome. For the purpose of illustration, we choose three biomarkers (FAM178A, IMAGE:813259 and UGP2), for which the missing rates are 31.3%, 45.5% and 26.3%, respectively. We conduct a logistic regression of 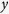 on the three biomarkers. In this analysis, *mi* and *mice* packages give error messages and MICE-RF approach is computationally very expensive. Therefore, we only use our two proposed MI methods (MICE-DURR and MICE-IURR) and the KNN-V and KNN-S methods in addition to the complete-case analysis. All 2107 biomarkers that do not have missing values are used to impute missing values in the three biomarkers.

Table 5 presents the results on logistic regression for the prostate cancer data. Based on our results, all three biomarkers become statistically significant after using our multiple imputation methods, except in one case that the p-value of UGP2 after MICE-DURR method is slightly larger than 0.05. In addition, in most cases, the estimates and p-values by MICE-DURR are consistent with those results by MICE-IURR. For example, the regression coefficients of biomarker (IMAGE:813259) after using two different multiple imputations (MICE-DURR and MICE-IURR) are 3.47 and 3.50, with p-values of 0.031 and 0.039, respectively.

## Discussion

We investigate two approaches for multiple imputation for general missing data patterns in the presence of high-dimensional data. Our numerical results demonstrate that the MICE-IURR approach performs better than the other imputation methods considered in terms of bias, whereas the MICE-DURR approach exhibits large bias and MSE. Of note, while MICE-RF leads to substantial bias in subsequent analysis of imputed data sets, it tends to yield smaller MSE than MICE-IURR due to smaller SD. In the case of comparing multiple imputation methods, it can be argued when one imputation method leads to substantial bias and hence incorrect inference in subsequent analysis of imputed data sets then whether this method yields smaller MSE may not be very relevant. Two data examples are used to further showcase the limitations of the existing imputation methods considered.

As alluded to earlier, while MICE is a flexible approach for handling different data types, its theoretical properties are not well-established. The specification of a set of conditional regression models may not be compatible with a joint distribution of the variables being imputed. Liu *et al*. (2013)^27^ established technical conditions for the convergence of the sequential conditional regression approach if the stationary joint distribution exists, which, however, may not happen in practice. Zhu and Raghunathan (2014)^28^ assessed theoretical properties of MI for both compatible and incompatible sequences of conditional regression models. However, their results are established for the missing data pattern where each subject may have missing values in at most one variable. One direction for future work is to extend these results to the settings of our interest.

## Additional Information

**How to cite this article**: Deng, Y. *et al*. Multiple Imputation for General Missing Data Patterns in the Presence of High-dimensional Data. *Sci. Rep.* **6**, 21689; doi: 10.1038/srep21689 (2016).

## Supplementary Material

Supplementary Information

## Figures and Tables

**Table 1:** Simulation results for estimating *β*_1_ = *β*_2_ = *β*_3_ = 1 in the presence of missing data based on 200 monte carlo data sets, where *n* = 100 and *ρ* = 0.1.

		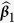					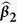					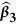				
		Bias	SE	SD	MSE	CR	Bias	SE	SD	MSE	CR	Bias	SE	SD	MSE	CR
*q* = 4	GS	0.010	0.519	0.484	0.233	0.960	−0.004	0.518	0.514	0.263	0.930	−0.003	0.522	0.507	0.255	0.960
	CC	−0.273	0.560	0.570	0.398	0.910	−0.277	0.561	0.556	0.385	0.915	−0.350	0.566	0.570	0.446	0.865
	MI-true	0.023	0.718	0.676	0.455	0.940	−0.013	0.719	0.721	0.517	0.920	−0.109	0.728	0.692	0.489	0.950
	*p* = 200															
	MICE-RF	−0.618	0.697	0.364	0.514	0.970	−0.469	0.699	0.331	0.329	1.000	−0.615	0.701	0.341	0.494	0.940
	KNN-V	−0.800	0.696	0.584	0.979	0.845	−0.369	0.700	0.552	0.440	0.975	−0.696	0.712	0.547	0.782	0.900
	KNN-S	−0.273	0.560	0.570	0.398	0.910	−0.277	0.561	0.556	0.385	0.915	−0.350	0.566	0.570	0.446	0.865
	MICE-DURR(Lasso)	−0.781	0.572	0.404	0.773	0.775	−0.543	0.567	0.431	0.479	0.895	−0.759	0.583	0.431	0.760	0.775
	MICE-DURR(EN)	−0.784	0.575	0.407	0.779	0.790	−0.534	0.568	0.428	0.467	0.895	−0.759	0.585	0.432	0.761	0.755
	MICE-DURR(Alasso)	−0.774	0.589	0.374	0.739	0.800	−0.557	0.581	0.384	0.458	0.925	−0.768	0.600	0.392	0.742	0.820
	MICE-IURR(Lasso)	−0.031	0.684	0.711	0.503	0.935	−0.017	0.681	0.697	0.484	0.920	−0.173	0.692	0.676	0.485	0.930
	MICE-IURR(EN)	−0.047	0.673	0.731	0.534	0.920	−0.009	0.670	0.703	0.491	0.910	−0.144	0.676	0.703	0.513	0.900
	MICE-IURR(Alasso)	−0.105	0.759	0.813	0.668	0.905	−0.181	0.751	0.794	0.660	0.910	−0.283	0.759	0.751	0.641	0.925
	*p* = 1000															
	MICE-DURR(Lasso)	−0.769	0.579	0.412	0.761	0.810	−0.568	0.576	0.474	0.547	0.885	−0.720	0.580	0.405	0.682	0.855
	MICE-DURR(EN)	−0.766	0.578	0.414	0.758	0.835	−0.558	0.581	0.465	0.526	0.900	−0.719	0.584	0.413	0.687	0.840
	MICE-DURR(Alasso)	−0.748	0.582	0.364	0.691	0.855	−0.576	0.583	0.441	0.525	0.905	−0.751	0.588	0.396	0.720	0.815
	MICE-IURR(Lasso)	0.021	0.683	0.780	0.605	0.885	−0.037	0.677	0.778	0.603	0.910	−0.168	0.689	0.714	0.536	0.910
	MICE-IURR(EN)	0.050	0.671	0.776	0.601	0.890	−0.049	0.671	0.779	0.606	0.900	−0.158	0.680	0.724	0.546	0.915
	MICE-IURR(Alasso)	−0.159	0.791	0.901	0.834	0.905	−0.245	0.777	0.849	0.777	0.905	−0.366	0.786	0.767	0.720	0.925
*q* = 20	GS	0.012	0.540	0.492	0.241	0.975	0.010	0.535	0.548	0.299	0.930	−0.008	0.542	0.508	0.256	0.965
	CC	−0.366	0.529	0.554	0.439	0.865	−0.359	0.526	0.517	0.395	0.905	−0.443	0.535	0.495	0.440	0.865
	MI-true	−0.097	0.715	0.627	0.400	0.950	−0.089	0.713	0.660	0.441	0.930	−0.133	0.728	0.683	0.482	0.950
	*p* = 200															
	MICE-RF	−0.468	0.730	0.429	0.402	0.975	−0.384	0.710	0.416	0.320	0.980	−0.447	0.725	0.412	0.369	0.980
	KNN-V	−0.412	0.684	0.527	0.446	0.945	−0.266	0.678	0.531	0.351	0.960	−0.389	0.691	0.544	0.445	0.945
	KNN-S	−0.366	0.529	0.554	0.439	0.865	−0.359	0.526	0.517	0.395	0.905	−0.443	0.535	0.495	0.440	0.865
	MICE-DURR(Lasso)	−0.494	0.675	0.464	0.458	0.960	−0.374	0.666	0.442	0.334	0.970	−0.443	0.678	0.442	0.390	0.970
	MICE-DURR(EN)	−0.499	0.677	0.480	0.478	0.965	−0.379	0.660	0.430	0.328	0.965	−0.435	0.679	0.448	0.389	0.975
	MICE-DURR(Alasso)	−0.488	0.685	0.475	0.462	0.945	−0.378	0.675	0.412	0.312	0.980	−0.457	0.693	0.423	0.387	0.985
	MICE-IURR(Lasso)	−0.058	0.695	0.714	0.510	0.915	0.058	0.671	0.732	0.536	0.875	−0.048	0.698	0.756	0.570	0.905
	MICE-IURR(EN)	−0.049	0.691	0.705	0.496	0.930	0.023	0.679	0.715	0.509	0.880	−0.041	0.697	0.743	0.551	0.910
	MICE-IURR(Alasso)	−0.214	0.714	0.735	0.583	0.905	−0.197	0.700	0.774	0.635	0.890	−0.309	0.719	0.760	0.671	0.880
	*p* = 1000															
	MICE-DURR(Lasso)	−0.435	0.677	0.470	0.409	0.970	−0.401	0.673	0.461	0.372	0.964	−0.471	0.679	0.437	0.412	0.976
	MICE-DURR(EN)	−0.443	0.673	0.476	0.422	0.964	−0.403	0.669	0.476	0.387	0.952	−0.451	0.678	0.463	0.416	0.982
	MICE-DURR(Alasso)	−0.434	0.678	0.475	0.412	0.976	−0.401	0.681	0.451	0.363	0.982	−0.474	0.683	0.433	0.411	0.970
	MICE-IURR(Lasso)	0.095	0.686	0.781	0.616	0.909	−0.019	0.687	0.858	0.732	0.885	−0.073	0.711	0.737	0.545	0.933
	MICE-IURR(EN)	0.082	0.689	0.776	0.606	0.897	−0.009	0.687	0.870	0.752	0.867	−0.069	0.705	0.749	0.562	0.915
	MICE-IURR(Alasso)	−0.212	0.739	0.758	0.615	0.927	−0.352	0.721	0.767	0.708	0.897	−0.394	0.732	0.745	0.707	0.885

Bias, mean bias; SE, mean standard error; SD, Monte Carlo standard deviation; MSE, mean square error; CR, coverage rate of 95% confidence interval; GS, gold standard; CC, complete-case; KNN-V, KNN by nearest variables; KNN-S, KNN by nearest subjects; MICE-DURR, MICE through direct use of regularized regressions; MICE-IURR, MICE through indirect use of regularized regressions; EN, elastic net; Alasso, adaptive lasso.

**Table 2:** Simulation results for estimating *β*_1_ = *β*_2_ = *β*_3_ = 1 in the presence of missing data based on 200 monte carlo data sets, where *n* = 100 and *ρ* = 0.5.

		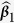					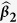					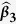				
		Bias	SE	SD	MSE	CR	Bias	SE	SD	MSE	CR	Bias	SE	SD	MSE	CR
*q* = 4	GS	0.007	0.515	0.478	0.228	0.970	−0.014	0.515	0.533	0.283	0.940	−0.037	0.519	0.496	0.246	0.970
	CC	−0.274	0.569	0.560	0.387	0.890	−0.272	0.570	0.588	0.418	0.910	−0.265	0.573	0.529	0.349	0.935
	MI-true	−0.068	0.687	0.687	0.474	0.925	0.010	0.698	0.717	0.512	0.915	−0.062	0.693	0.685	0.470	0.925
	*p* = 200															
	MICE-RF	−0.628	0.747	0.352	0.518	0.960	−0.355	0.711	0.371	0.263	0.980	−0.510	0.725	0.349	0.382	0.975
	KNN-V	−0.930	0.708	0.545	1.160	0.800	−0.242	0.711	0.535	0.343	0.980	−0.655	0.720	0.557	0.738	0.925
	KNN-S	−0.274	0.569	0.560	0.387	0.890	−0.272	0.570	0.588	0.418	0.910	−0.265	0.573	0.529	0.349	0.935
	MICE-DURR(Lasso)	−0.853	0.548	0.407	0.892	0.720	−0.475	0.544	0.384	0.373	0.925	−0.688	0.546	0.418	0.647	0.860
	MICE-DURR(EN)	−0.852	0.548	0.412	0.894	0.695	−0.474	0.541	0.386	0.372	0.935	−0.688	0.550	0.411	0.641	0.845
	MICE-DURR(Alasso)	−0.844	0.566	0.349	0.834	0.785	−0.486	0.563	0.351	0.359	0.945	−0.701	0.570	0.374	0.631	0.870
	MICE-IURR(Lasso)	−0.094	0.668	0.690	0.483	0.930	−0.080	0.669	0.729	0.536	0.885	−0.068	0.661	0.703	0.496	0.925
	MICE-IURR(EN)	−0.092	0.653	0.701	0.498	0.935	−0.074	0.667	0.724	0.527	0.910	−0.069	0.647	0.705	0.499	0.915
	MICE-IURR(Alasso)	−0.091	0.720	0.744	0.559	0.940	−0.076	0.710	0.790	0.626	0.880	−0.123	0.719	0.761	0.592	0.920
	*p* = 1000															
	MICE-DURR(Lasso)	−0.809	0.560	0.392	0.807	0.715	−0.534	0.558	0.445	0.482	0.905	−0.713	0.568	0.437	0.698	0.825
	MICE-DURR(EN)	−0.806	0.560	0.398	0.808	0.710	−0.530	0.560	0.458	0.490	0.920	−0.711	0.568	0.433	0.693	0.815
	MICE-DURR(Alasso)	−0.803	0.567	0.354	0.770	0.755	−0.544	0.565	0.409	0.463	0.905	−0.730	0.577	0.379	0.676	0.840
	MICE-IURR(Lasso)	0.058	0.677	0.836	0.698	0.860	−0.040	0.676	0.811	0.656	0.890	−0.197	0.677	0.752	0.601	0.905
	MICE-IURR(EN)	0.073	0.664	0.808	0.655	0.850	−0.041	0.667	0.800	0.638	0.890	−0.192	0.670	0.739	0.581	0.885
	MICE-IURR(Alasso)	−0.036	0.793	0.912	0.829	0.865	−0.209	0.783	0.852	0.765	0.885	−0.335	0.779	0.792	0.737	0.910
*q* = 20	GS	0.014	0.521	0.500	0.249	0.950	−0.008	0.523	0.550	0.301	0.920	−0.023	0.527	0.506	0.255	0.970
	CC	−0.352	0.525	0.536	0.410	0.855	−0.357	0.529	0.541	0.418	0.875	−0.387	0.533	0.520	0.419	0.890
	MI-true	−0.077	0.689	0.593	0.356	0.965	−0.047	0.688	0.675	0.456	0.920	−0.092	0.692	0.628	0.401	0.965
	*p* = 200															
	MICE-RF	−0.390	0.717	0.427	0.333	0.990	−0.286	0.708	0.466	0.298	0.985	−0.412	0.721	0.382	0.315	0.995
	KNN-V	−0.450	0.685	0.530	0.482	0.950	−0.214	0.697	0.561	0.359	0.970	−0.441	0.703	0.469	0.413	0.980
	KNN-S	−0.352	0.525	0.536	0.410	0.855	−0.357	0.529	0.541	0.418	0.875	−0.387	0.533	0.520	0.419	0.890
	MICE-DURR(Lasso)	−0.491	0.631	0.451	0.444	0.955	−0.329	0.633	0.486	0.343	0.970	−0.532	0.644	0.421	0.459	0.975
	MICE-DURR(EN)	−0.489	0.631	0.455	0.445	0.920	−0.340	0.627	0.486	0.351	0.970	−0.518	0.641	0.401	0.428	0.970
	MICE-DURR(Alasso)	−0.481	0.655	0.425	0.412	0.940	−0.348	0.653	0.452	0.325	0.975	−0.529	0.668	0.377	0.422	0.960
	MICE-IURR(Lasso)	−0.022	0.678	0.729	0.530	0.905	−0.024	0.684	0.752	0.563	0.870	−0.057	0.683	0.689	0.475	0.905
	MICE-IURR(EN)	−0.023	0.666	0.695	0.481	0.920	−0.022	0.670	0.730	0.531	0.855	−0.036	0.675	0.674	0.453	0.925
	MICE-IURR(Alasso)	−0.144	0.731	0.721	0.539	0.910	−0.103	0.719	0.764	0.591	0.895	−0.181	0.720	0.718	0.546	0.955
	*p* = 1000															
	MICE-DURR(Lasso)	−0.436	0.640	0.415	0.361	0.978	−0.399	0.628	0.490	0.396	0.956	−0.458	0.640	0.393	0.363	0.989
	MICE-DURR(EN)	−0.459	0.631	0.437	0.399	0.978	−0.375	0.626	0.492	0.380	0.967	−0.460	0.636	0.407	0.375	1.000
	MICE-DURR(Alasso)	−0.450	0.651	0.419	0.376	0.978	−0.387	0.642	0.482	0.380	0.978	−0.487	0.655	0.384	0.382	0.989
	MICE-IURR(Lasso)	0.012	0.682	0.750	0.556	0.900	0.004	0.660	0.712	0.502	0.956	−0.079	0.687	0.630	0.398	0.933
	MICE-IURR(EN)	0.020	0.672	0.738	0.539	0.878	0.018	0.662	0.725	0.521	0.911	−0.086	0.653	0.660	0.438	0.933
	MICE-IURR(Alasso)	−0.204	0.766	0.767	0.624	0.878	−0.135	0.745	0.778	0.617	0.889	−0.318	0.737	0.666	0.540	0.911

Bias, mean bias; SE, mean standard error; SD, Monte Carlo standard deviation; MSE, mean square error; CR, coverage rate of 95% confidence interval; GS, gold standard; CC, complete-case; KNN-V, KNN by nearest variables; KNN-S, KNN by nearest subjects; MICE-DURR, MICE through direct use of regularized regressions; MICE-IURR, MICE through indirect use of regularized regressions; EN, elastic net; Alasso, adaptive lasso.

**Table 3:** Simulation results for estimating *β*_1_ = *β*_2_ = *β*_3_ = 1 in the presence of missing data based on 200 monte carlo data sets, where *n* = 100 and *ρ* = 0.9.

		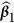					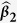					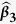				
		Bias	SE	SD	MSE	CR	Bias	SE	SD	MSE	CR	Bias	SE	SD	MSE	CR
*q* = 4	GS	0.029	0.515	0.476	0.226	0.960	0.003	0.512	0.521	0.270	0.945	0.008	0.517	0.489	0.238	0.970
	CC	−0.219	0.583	0.609	0.417	0.905	−0.245	0.585	0.560	0.371	0.925	−0.242	0.582	0.582	0.395	0.945
	MI-true	−0.012	0.711	0.699	0.487	0.935	−0.027	0.710	0.715	0.509	0.915	−0.007	0.701	0.685	0.467	0.940
	*p* = 200															
	MICE-RF	−0.483	0.721	0.358	0.361	0.980	−0.277	0.704	0.376	0.218	1.000	−0.372	0.722	0.380	0.283	0.975
	KNN-V	−1.034	0.747	0.584	1.408	0.765	−0.113	0.741	0.596	0.366	0.990	−0.676	0.758	0.630	0.852	0.895
	KNN-S	−0.219	0.583	0.609	0.417	0.905	−0.245	0.585	0.560	0.371	0.925	−0.242	0.582	0.582	0.395	0.945
	MICE-DURR(Lasso)	−0.857	0.547	0.435	0.922	0.670	−0.549	0.532	0.417	0.475	0.905	−0.643	0.545	0.441	0.606	0.835
	MICE-DURR(EN)	−0.862	0.548	0.443	0.939	0.670	−0.534	0.537	0.420	0.461	0.880	−0.649	0.550	0.438	0.612	0.815
	MICE-DURR(Alasso)	−0.841	0.571	0.409	0.874	0.745	−0.525	0.564	0.377	0.417	0.935	−0.677	0.576	0.396	0.615	0.855
	MICE-IURR(Lasso)	−0.049	0.676	0.701	0.491	0.915	−0.047	0.671	0.694	0.481	0.920	−0.027	0.669	0.702	0.492	0.920
	MICE-IURR(EN)	−0.028	0.671	0.716	0.511	0.915	−0.059	0.676	0.707	0.501	0.910	−0.052	0.677	0.706	0.498	0.935
	MICE-IURR(Alasso)	−0.005	0.684	0.710	0.502	0.920	−0.024	0.685	0.727	0.526	0.915	−0.031	0.679	0.710	0.503	0.935
	*p* = 1000															
	MICE-DURR(Lasso)	−0.787	0.556	0.399	0.778	0.740	−0.494	0.538	0.405	0.408	0.920	−0.688	0.559	0.375	0.613	0.845
	MICE-DURR(EN)	−0.793	0.554	0.414	0.799	0.715	−0.490	0.534	0.410	0.407	0.900	−0.681	0.562	0.373	0.603	0.840
	MICE-DURR(Alasso)	−0.801	0.572	0.363	0.772	0.775	−0.496	0.557	0.369	0.382	0.930	−0.705	0.577	0.339	0.611	0.880
	MICE-IURR(Lasso)	−0.014	0.682	0.744	0.550	0.930	−0.058	0.672	0.763	0.582	0.915	−0.144	0.680	0.709	0.521	0.920
	MICE-IURR(EN)	0.009	0.679	0.729	0.529	0.935	−0.062	0.667	0.744	0.555	0.915	−0.155	0.672	0.702	0.515	0.930
	MICE-IURR(Alasso)	−0.029	0.729	0.739	0.545	0.935	−0.024	0.716	0.818	0.666	0.895	−0.130	0.715	0.760	0.592	0.910
*q* = 20	GS	0.018	0.514	0.478	0.228	0.945	−0.010	0.512	0.519	0.268	0.945	−0.003	0.515	0.488	0.237	0.975
	CC	−0.260	0.570	0.562	0.382	0.930	−0.295	0.566	0.533	0.370	0.930	−0.304	0.568	0.537	0.379	0.925
	MI-true	−0.056	0.681	0.561	0.316	0.975	−0.075	0.671	0.590	0.352	0.980	−0.009	0.667	0.566	0.319	0.960
	*p* = 200															
	MICE-RF	−0.334	0.747	0.361	0.241	0.995	−0.153	0.707	0.362	0.154	1.000	−0.269	0.756	0.398	0.230	1.000
	KNN-V	−0.798	0.749	0.555	0.943	0.895	−0.108	0.739	0.555	0.318	1.000	−0.506	0.757	0.569	0.578	0.960
	KNN-S	−0.260	0.570	0.562	0.382	0.930	−0.295	0.566	0.533	0.370	0.930	−0.304	0.568	0.537	0.379	0.925
	MICE-DURR(Lasso)	−0.690	0.576	0.412	0.645	0.880	−0.392	0.557	0.404	0.316	0.945	−0.575	0.574	0.406	0.495	0.930
	MICE-DURR(EN)	−0.679	0.579	0.420	0.636	0.880	−0.379	0.556	0.404	0.306	0.955	−0.591	0.572	0.414	0.520	0.920
	MICE-DURR(Alasso)	−0.668	0.616	0.382	0.592	0.915	−0.393	0.593	0.391	0.306	0.970	−0.611	0.611	0.381	0.519	0.945
	MICE-IURR(Lasso)	−0.051	0.665	0.646	0.418	0.935	−0.031	0.666	0.678	0.458	0.910	0.007	0.660	0.658	0.431	0.950
	MICE-IURR(EN)	−0.031	0.665	0.645	0.415	0.935	−0.057	0.662	0.660	0.436	0.930	0.006	0.659	0.655	0.427	0.945
	MICE-IURR(Alasso)	−0.035	0.668	0.691	0.476	0.915	−0.063	0.667	0.660	0.437	0.935	−0.003	0.667	0.684	0.466	0.935
	*p* = 1000															
	MICE-DURR(Lasso)	−0.738	0.582	0.417	0.717	0.850	−0.408	0.569	0.417	0.340	0.955	−0.531	0.586	0.444	0.478	0.905
	MICE-DURR(EN)	−0.729	0.587	0.416	0.704	0.860	−0.416	0.569	0.421	0.350	0.945	−0.526	0.587	0.443	0.472	0.925
	MICE-DURR(Alasso)	−0.723	0.614	0.384	0.670	0.920	−0.433	0.600	0.374	0.327	0.950	−0.540	0.612	0.401	0.451	0.945
	MICE-IURR(Lasso)	0.055	0.687	0.725	0.526	0.905	−0.057	0.676	0.770	0.593	0.890	−0.124	0.676	0.735	0.553	0.895
	MICE-IURR(EN)	0.052	0.675	0.746	0.556	0.900	−0.053	0.673	0.782	0.611	0.910	−0.113	0.669	0.751	0.573	0.915
	MICE-IURR(Alasso)	−0.003	0.742	0.769	0.589	0.930	−0.105	0.728	0.803	0.653	0.920	−0.082	0.732	0.792	0.631	0.915

Bias, mean bias; SE, mean standard error; SD, Monte Carlo standard deviation; MSE, mean square error; CR, coverage rate of 95% confidence interval; GS, gold standard; CC, complete-case; KNN-V, KNN by nearest variables; KNN-S, KNN by nearest subjects; MICE-DURR, MICE through direct use of regularized regressions; MICE-IURR, MICE through indirect use of regularized regressions; EN, elastic net; Alasso, adaptive lasso.

**Table 4:** Regression coefficients estimates of the Georgia stroke registry data.

Characteristics	CC	mi	mice	MICE-RF	MICE-DURR	MICE-IURR
NIH stroke score	−1.95 (<0.001)	−2.04 (0.010)	−6.07 (<0.001)	−5.01 (<0.001)	−1.10 (0.236)	−1.00 (0.176)
	[−2.7, −1.2]	[−3.48, −0.6]	[−8.5, −3.64]	[−6.38, −3.63]	[−3.04, 0.84]	[−2.5, 0.49]
EMS pre-notification	−3.17 (0.590)	−19.83 (0.043)	−0.82 (0.957)	−5.23 (0.604)	−2.22 (0.819)	−5.92 (0.658)
	[−14.69, 8.35]	[−38.7, −0.96]	[−34.04, 32.4]	[−25.35, 14.9]	[−21.59, 17.14]	[−34.44, 22.59]
Serum total lipid	−0.07 (0.201)	−0.47 (<0.001)	−0.52 (<0.001)	−0.36 (<0.001)	−0.26 (0.036)	−0.26 (0.005)
	[−0.18, 0.04]	[−0.62, −0.32]	[−0.7, −0.33]	[−0.53, −0.19]	[−0.49, −0.02]	[−0.43, −0.08]
Age	0.02 (0.936)	−0.87 (0.022)	−0.78 (0.042)	−0.71 (0.061)	−0.76 (0.045)	−0.80 (0.037)
	[−0.51, 0.56]	[−1.62, −0.12]	[−1.53, −0.03]	[−1.46, 0.03]	[−1.51, −0.02]	[−1.54, −0.05]
Male(referent: female)	5.33 (0.372)	21.20 (0.013)	24.83 (0.004)	19.15 (0.025)	16.57 (0.053)	16.54 (0.053)
	[−6.37, 17.02]	[4.41, 37.98]	[8, 41.66]	[2.41, 35.89]	[−0.24, 33.38]	[−0.19, 33.27]
White(referent: African American)	−16.64 (0.007)	−14.44 (0.107)	−20.07 (0.028)	−17.64 (0.048)	−14.11 (0.114)	−13.85 (0.121)
	[−28.82, −4.45]	[−32.01, 3.14]	[−37.98, −2.15]	[−35.16, −0.12]	[−31.62, 3.41]	[−31.34, 3.65]
Health insurance by medicare	−4.07 (0.617)	−24.95 (0.032)	−24.89 (0.032)	−24.35 (0.036)	−24.36 (0.036)	−24.05 (0.038)
	[−20.04, 11.9]	[−47.72, −2.19]	[−47.7, −2.08]	[−47.13, −1.57]	[−47.11, −1.6]	[−46.8, −1.3]
Arrive in the daytime	4.94 (0.420)	−23.10 (0.011)	−24.64 (0.006)	−10.27 (0.275)	−18.97 (0.043)	−18.41 (0.045)
	[−7.07, 16.96]	[−40.89, −5.31]	[−42.35, −6.94]	[−28.74, 8.2]	[−37.38, −0.56]	[−36.39, −0.42]
NPO	8.37 (0.393)	58.79 (0.001)	121.04 (<0.001)	81.03 (0.001)	39.98 (0.006)	43.31 (0.001)
	[−10.84, 27.58]	[26.65, 90.93]	[76.21, 165.88]	[39.6, 122.45]	[11.87, 68.08]	[17.25, 69.37]
History of stroke	−2.57 (0.695)	−36.55 (0.001)	−34.10 (0.002)	−28.99 (0.009)	−31.96 (0.008)	−33.24 (0.002)
	[−15.43, 10.29]	[−57.15, −15.96]	[−55.86, −12.35]	[−50.72, −7.27]	[−55.52, −8.41]	[−54.47, −12]
History of TIA	−16.30 (0.097)	−64.53 (<0.001)	−89.47 (<0.001)	−64.94 (<0.001)	−62.39 (<0.001)	−60.34 (<0.001)
	[−35.54, 2.94]	[−95.93, −33.13]	[−123.95, −55]	[−97.47, −32.41]	[−96.59, −28.19]	[−92.47, −28.2]
History of cardiac valve prosthesis	−27.25 (0.349)	89.28 (0.016)	136.94 (<0.001)	126.22 (0.022)	103.18 (0.008)	104.15 (0.007)
	[−84.27, 29.78]	[16.98, 161.59]	[79.4, 194.48]	[19.67, 232.77]	[27.19, 179.16]	[28.31, 179.98]
Family history of stroke	−17.33 (0.406)	−85.07 (0.014)	−51.10 (0.078)	−82.91 (0.022)	−79.79 (0.028)	−76.82 (0.034)
	[−58.18, 23.51]	[−153.23, −16.92]	[−107.91, 5.72]	[−153.95, −11.87]	[−150.94, −8.65]	[−147.91, −5.74]

KNN-V and KNN-S are not included because of errors. NPO, nil per os, Latin for “nothing by mouth”, a medical instruction to withhold oral intake of food and fluids from a patient. P-value, (); 95% confidence interval, [].

**Table 5:** Regression coefficients estimates of the prostate cancer data.

Biomarkers	CC	KNN-V	KNN-S	MICE-DURR	MICE-IURR	Missing-rate
FAM178A	5.80 (<0.119)	5.62 (<0.001)	5.33 (0.001)	4.43(0.003)	4.70(0.002)	31.3%
	[−1.49, 13.09]	[2.62, 8.62]	[2.31, 8.35]	[1.61, 7.25]	[1.76, 7.64]	
IMAGE:813259	6.03 (0.151)	4.20 (0.009)	4.43 (0.016)	3.47 (0.031)	3.50 (0.039)	45.5%
	[−2.2, 14.26]	[1.06, 7.34]	[0.82, 8.04]	[0.37, 6.57]	[0.23, 6.77]	
UGP2	−2.57 (0.386)	−3.44 (0.021)	−3.45 (0.021)	−2.32 (0.067)	−3.15 (0.025)	26.3%
	[−8.37, 3.23]	[−6.36, −0.52]	[−6.37, −0.53]	[−4.77, 0.13]	[−5.85, −0.45]	

MICE-RF is not included because of errors. P-value, (); 95% confidence interval, [].
