# Supplementary material for: Multiple Imputation for General Missing Data Patterns in the Presence of High-dimensional Data
Source: Sci Rep. 2016 Feb 12;6:21689. doi: 10.1038/srep21689 (PMC4751511; doi:10.1038/srep21689)
Supplement: Supplementary Information [file srep21689-s1.pdf]

Multiple Imputation for General Missing Data Patterns in the Presence of High-dimensional Data

Yi Deng[1], Changgee Chang[1], Moges Ido[2], and Qi Long[1,*]

[1]Department of Biostatistics and Bioinformatics, Emory University, Atlanta, 30322, USA
[2]Georgia Department of Public Health, 30303, USA
[*]Correspondence: qlong@emory.edu

Supplementary Methods

Method S1: Details of MICE-DURR for three types of data
Method S2: Details of MICE-IURR for three types of data

Method S1: Details of MICE-DURR for three types of data

We start the iterative procedure with some initial values. For example, all the elements in $\mathbf{z}_{mis,j}$ are filled in with the average of the observed values of $\mathbf{z}_j$ $(j = 1, 2, ..., l)$. Define the corresponding initial completed dataset as $\mathbf{Z}^{(0)}$.

In the m-th iteration:

(i) If $\mathbf{z}_j$ follows a Gaussian distribution, the model is

$$\mathbf{z}^*_{j,obs} = \theta_{0,j}\mathbf{1}_{r^*_j} + \mathbf{W}^{*(m)}_{j,obs}\theta_j + \varepsilon_j, \tag{1}$$

where r^*_j is the number of cases with observed $\mathbf{z}^*_j$ and $\varepsilon_j \sim N(0, \sigma^2_j \mathbf{I}_{r^*_j})$.
A regularized regression method is used to fit model (1). The parameter estimates can be obtained as follows:

$$(\widehat{\theta}^{(m)}_{0,j}, \widehat{\theta}^{(m)}_j) = \underset{(\theta_{0,j},\theta_j)}{\text{argmin}}[-\ell(\theta_{0,j}, \theta_j; \mathbf{z}^*_{j,obs}, \mathbf{W}^{*(m)}_{j,obs}) + P_\lambda(\theta_j)]$$

Where $P_\lambda(\theta_j)$ is a regularization function. We consider the mean of squared residuals as an estimate of σ^2_j, denoted by $\widehat{\sigma}^{2(m)}_j$.

$\mathbf{z}_{j,mis}$ is predicted with $\mathbf{z}^{(m)}_{j,mis}$ by drawing randomly from the predictive distribution $N(\widehat{\theta}^{(m)}_{0,j}\mathbf{1}_{n-r_j} + \mathbf{W}^{(m)}_{j,mis}\widehat{\theta}^{(m)}_j, \widehat{\sigma}^{2(m)}_j \mathbf{I}_{n-r_j})$.
Let $\mathbf{z}^{(m)}_j = (\mathbf{z}^{(m)}_{j,mis}, \mathbf{z}_{j,obs})$.

(ii) If $\mathbf{z}_j$ follows a Bernoulli distribution, the model is

$$logit(\mathbf{z}^*_{j,obs} = 1|\mathbf{W}^{*(m)}_{j,obs}) = \theta_{0,j}\mathbf{1}_{r^*_j} + \mathbf{W}^{*(m)}_{j,obs}\theta_j, \tag{2}$$

A regularized regression method is used to fit model (2). The parameter estimates can be obtained as follows:

$$(\widehat{\theta}^{(m)}_{0,j}, \widehat{\theta}^{(m)}_j) = \underset{(\theta_{0,j},\theta_j)}{\text{argmin}}[-\ell(\theta_{0,j}, \theta_j; \mathbf{z}^*_{j,obs}, \mathbf{W}^{*(m)}_{j,obs}) + P_\lambda(\theta_j)]$$

Where $P_\lambda(\theta_j)$ is a regularization function.

$\mathbf{z}_{j,mis}$ is predicted with $\mathbf{z}^{(m)}_{j,mis}$ by drawing randomly from the predictive distribution $Bernoulli(\frac{exp(\widehat{\theta}^{(m)}_{0,j}\mathbf{1}_{n-r_j} + \mathbf{W}^{(m)}_{j,mis}\widehat{\theta}^{(m)}_j)}{1+exp(\widehat{\theta}^{(m)}_{0,j}\mathbf{1}_{n-r_j} + \mathbf{W}^{(m)}_{j,mis}\widehat{\theta}^{(m)}_j)})$.
Let $\mathbf{z}^{(m)}_j = (\mathbf{z}^{(m)}_{j,mis}, \mathbf{z}_{j,obs})$.

(iii) If $\mathbf{z}_j$ follows a Poisson distribution, the model is

$$log(\mathbf{E}[\mathbf{z}^*_{j,obs}|\mathbf{W}^{*(m)}_{j,obs}]) = \theta_{0,j}\mathbf{1}_{r^*_j} + \mathbf{W}^{*(m)}_{j,obs}\theta_j, \tag{3}$$

A regularized regression method is used to fit model (3). The parameter estimates can be obtained as follows:

$$(\widehat{\theta}^{(m)}_{0,j}, \widehat{\theta}^{(m)}_j) = \underset{(\theta_{0,j},\theta_j)}{\operatorname{argmin}}[-\ell(\theta_{0,j},\theta_j;\mathbf{z}^*_{j,obs},\mathbf{W}^{*(m)}_{j,obs}) + P_\lambda(\theta_j)]$$

Where $P_\lambda(\theta_j)$ is a regularization function.
$\mathbf{z}_{j,mis}$ is predicted with $\mathbf{z}^{(m)}_{j,mis}$ by drawing randomly from the predictive distribution $Poisson(exp(\widehat{\theta}^{(m)}_{0,j}\mathbf{1}_{n-r_j} + \mathbf{W}^{(m)}_{j,mis}\widehat{\theta}^{(m)}_j))$. Let $\mathbf{z}^{(m)}_j = (\mathbf{z}^{(m)}_{j,mis}, \mathbf{z}_{j,obs})$.

We denote the updated data set after the m-th interation by $\mathbf{Z}^{(m)}$ and repeat the procedures iteratively. After the algorithm converges, the last M imputed data sets after appropriate thinning are chosen for subsequent standard complete-data analysis.

Method S2: Details of MICE-IURR for three types of data

We start the iterative procedure with some initial values. For example, all the elements in $\mathbf{z}_{mis,j}$ are filled in with the average of the observed values of $\mathbf{z}_j$ $(j = 1,2,...,l)$. Define the corresponding initial completed dataset as $\mathbf{Z}^{(0)}$.

In the m-th iteration:

(i) If $\mathbf{z}_j$ follows a Gaussian distribution, we use a regularized regression method to fit a multiple linear regression model regarding $\mathbf{z}_{j,obs}$ as the outcome variable and $\mathbf{W}^{(m)}_{j,obs}$ as the predictor variable, and identify the active set, $\widehat{\mathscr{S}}^{(m)}_j$. Let $\mathbf{W}_{\widehat{\mathscr{S}}^{(m)}_j}$ denote the subset of $\mathbf{W}^{(m)}_j$ that only contains the active set. Correspondingly, denote two components of $\mathbf{W}_{\widehat{\mathscr{S}}^{(m)}_j}$ by $\mathbf{W}_{\widehat{\mathscr{S}}^{(m)}_j,mis}$ and $\mathbf{W}_{\widehat{\mathscr{S}}^{(m)}_j,obs}$. Then the model is

$$\mathbf{z}_{j,obs} = \theta_{0,j}\mathbf{1}_{r_j} + \mathbf{W}_{\widehat{\mathscr{S}}^{(m)}_j,obs}\theta_j + \varepsilon_j, \tag{4}$$

where $\varepsilon_j \sim N(0,\sigma^2_j\mathbf{I}_{r_j})$ and $\mathbf{1}_{r_j}$ is a vector of length r_j with all entries one.
Approximate the distribution of $(\theta_{0,j},\theta_j,\sigma^2_j)$ by using a standard inference procedure such as maximum likelihood.

$$(\theta_{0,j},\theta_j,\sigma^2_j)' \sim N(\widehat{\theta}^{(m)}_{MLE}, \widehat{\Sigma}^{(m)}_{MLE})$$

Where $\widehat{\theta}^{(m)}_{MLE}$ is the MLE of parameters in model (4) and $\widehat{\Sigma}^{(m)}_{MLE}$ is the variance-covariance matrix of the estimated parameters.
Generate a prediction for $\mathbf{z}_{j,mis}$: randomly draw $(\widehat{\theta}^{(m)}_{0,j}, \widehat{\theta}^{(m)}_j, \widehat{\sigma}^{2(m)}_j)$ from $N(\widehat{\theta}^{(m)}_{MLE}, \widehat{\Sigma}^{(m)}_{MLE})$, and predict $\mathbf{z}_{j,mis}$ with $\mathbf{z}^{(m)}_{j,mis}$ by drawing randomly from the predictive distribution $N(\widehat{\theta}^{(m)}_{0,j}\mathbf{1}_{n-r_j} + \mathbf{W}_{\widehat{\mathscr{S}}^{(m)}_j,mis}\widehat{\theta}^{(m)}_j, \widehat{\sigma}^{2(m)}_j\mathbf{I}_{n-r_j})$. Let $\mathbf{z}^{(m)}_j = (\mathbf{z}^{(m)}_{j,mis}, \mathbf{z}_{j,obs})$.

(ii) If $\mathbf{z}_j$ follows a Bernoulli distribution, we use a regularized regression method to fit a multiple linear regression model regarding $\mathbf{z}_{j,obs}$ as the outcome variable and $\mathbf{W}^{(m)}_{j,obs}$ as the predictor variable, and identify the active set, $\widehat{\mathscr{S}}^{(m)}_j$. Let $\mathbf{W}_{\widehat{\mathscr{S}}^{(m)}_j}$ denote the subset of $\mathbf{W}^{(m)}_j$ that only contains the active set. Correspondingly, denote two components of $\mathbf{W}_{\widehat{\mathscr{S}}^{(m)}_j}$ by $\mathbf{W}_{\widehat{\mathscr{S}}^{(m)}_j,mis}$ and $\mathbf{W}_{\widehat{\mathscr{S}}^{(m)}_j,obs}$. Then the model is

$$logit(\Pr(\mathbf{z}_{j,obs} = 1|\mathbf{W}_{\widehat{\mathscr{S}}^{(m)}_j,obs})) = \theta_{0,j}\mathbf{1}_{r_j} + \mathbf{W}_{\widehat{\mathscr{S}}^{(m)}_j,obs}\theta_j, \tag{5}$$

Approximate the distribution of $(\theta_{0,j},\theta_j)$ by using a standard inference procedure such as maximum likelihood.

$$(\theta_{0,j},\theta_j)' \sim N(\widehat{\theta}^{(m)}_{MLE}, \widehat{\Sigma}^{(m)}_{MLE})$$

Where $\widehat{\theta}^{(m)}_{MLE}$ is the MLE of parameters in model (5) and $\widehat{\Sigma}^{(m)}_{MLE}$ is the variance-covariance matrix of the estimated parameters.

Generate a prediction for $\mathbf{z}_{j,mis}$: randomly draw $(\widehat{\theta}^{(m)}_{0,j}, \widehat{\theta}^{(m)}_{j})$ from $N(\widehat{\theta}^{(m)}_{MLE}, \widehat{\Sigma}^{(m)}_{MLE})$, and predict $\mathbf{z}_{j,mis}$ with $\mathbf{z}^{(m)}_{j,mis}$ by drawing randomly from the predictive distribution $Bernoulli(\frac{exp(\widehat{\theta}^{(m)}_{0,j}\mathbf{1}_{n-r_j}+\mathbf{W}_{\widehat{\mathscr{S}}^{(m)}_j,mis}\widehat{\theta}^{(m)}_j)}{1+exp(\widehat{\theta}^{(m)}_{0,j}\mathbf{1}_{n-r_j}+\mathbf{W}_{\widehat{\mathscr{S}}^{(m)}_j,mis}\widehat{\theta}^{(m)}_j)})$. Let $\mathbf{z}^{(m)}_j = (\mathbf{z}^{(m)}_{j,mis}, \mathbf{z}_{j,obs})$.

(iii) If $\mathbf{z}_j$ follows a Poisson distribution, we use a regularized regression method to fit a multiple linear regression model regarding $\mathbf{z}_{j,obs}$ as the outcome variable and $\mathbf{W}^{(m)}_{j,obs}$ as the predictor variable, and identify the active set, $\widehat{\mathscr{S}}^{(m)}_j$. Let $\mathbf{W}_{\widehat{\mathscr{S}}^{(m)}_j}$ denote the subset of $\mathbf{W}^{(m)}_j$ that only contains the active set. Correspondingly, denote two components of $\mathbf{W}_{\widehat{\mathscr{S}}^{(m)}_j}$ by $\mathbf{W}_{\widehat{\mathscr{S}}^{(m)}_j,mis}$ and $\mathbf{W}_{\widehat{\mathscr{S}}^{(m)}_j,obs}$. Then the model is

$$log(\mathbf{E}[\mathbf{z}_{j,obs}|\mathbf{W}_{\widehat{\mathscr{S}}^{(m)}_j,obs}]) = \theta_{0,j}\mathbf{1}_{r_j} + \mathbf{W}_{\widehat{\mathscr{S}}^{(m)}_j,obs}\theta_j, \tag{6}$$

Approximate the distribution of $(\theta_{0,j}, \theta_j)$ by using a standard inference procedure such as maximum likelihood.

$$(\theta_{0,j}, \theta_j)' \sim N(\widehat{\theta}^{(m)}_{MLE}, \widehat{\Sigma}^{(m)}_{MLE})$$

Where $\widehat{\theta}^{(m)}_{MLE}$ is the MLE of parameters in model (6) and $\widehat{\Sigma}^{(m)}_{MLE}$ is the variance-covariance matrix of the estimated parameters.

Generate a prediction for $\mathbf{z}_{j,mis}$: randomly draw $(\widehat{\theta}^{(m)}_{0,j}, \widehat{\theta}^{(m)}_{j})$ from $N(\widehat{\theta}^{(m)}_{MLE}, \widehat{\Sigma}^{(m)}_{MLE})$, and predict $\mathbf{z}_{j,mis}$ with $\mathbf{z}^{(m)}_{j,mis}$ by drawing randomly from the predictive distribution $Poisson(exp(\widehat{\theta}^{(m)}_{0,j}\mathbf{1}_{n-r_j} + \mathbf{W}_{\widehat{\mathscr{S}}^{(m)}_j,mis}\widehat{\theta}^{(m)}_j))$. Let $\mathbf{z}^{(m)}_j = (\mathbf{z}^{(m)}_{j,mis}, \mathbf{z}_{j,obs})$.

We denote the updated data set after the m-th interation by $\mathbf{Z}^{(m)}$ and repeat the procedures iteratively. After the algorithm converges, the last M imputed data sets after appropriate thinning are chosen for subsequent standard complete-data analysis.
